# Establishing Initial Guidelines for Hospice Caregiver Outreach and Recruitment

**DOI:** 10.1093/geroni/igaf122.3579

**Published:** 2025-12-31

**Authors:** Felix Vasquez, Elizabeth Luth, Veerawat Phongtankuel, Kira Sheldon

**Affiliations:** VNS Health, Brooklyn, New York, United States; Rutgers University, New Brunswick, New Jersey, United States; Weill Cornell Medicine, New York, New York, United States; Rutgers University-New Brunswick, New Brunswick, New Jersey, United States

## Abstract

Caregiver recruitment is historically challenging in home hospice research, and there are few established best practices recruiting from this population. This analysis aimed to identify trends in home hospice caregiver recruitment to aide investigators in planning studies focused on enrolling hospice caregivers. Recruitment data was compiled from two intervention studies (EDITH-HC and I-HoME) intended to improve the quality of care for home hospice patients and their caregivers and reduce caregiver burden. Caregivers contacted for participation from both studies were aggregated, and 144 of 461 (33%) eligible caregivers enrolled. Recruitment began 14-days after the start of care (SOC) OASIS assessment was completed. We found that thirty-two percent (32%, n = 111 of 342) caregivers who were contacted between 14-90 days from SOC enrolled. Eighty-one percent (81%, n = 90 of 111) of caregivers who enrolled within 14-90 days from SOC also enrolled within the first two recruitment calls. Out of the 1312 calls made, the highest percentage of caregivers answered calls and enrolled during the early afternoon (12pm-3pm: 31% n = 22 of 71), followed by evening (6-8pm: 31%, n = 10 of 32), morning (9am-12pm: 29%, n = 15 of 51), and late afternoon (3pm-6pm: 19%, n = 81 of 436). Eighteen percent (18%, n = 232 of 1312) of calls are missing the time-of-day caregivers were contacted. Recommendations to improve future recruitment practices include calling at least twice, within the first 14-90 days of hospice enrollment, and having the flexibility to call at all times of the day. These findings provide initial guidelines for maximizing recruitment among the hard-to-reach population of hospice caregivers.

